# Hope and its associations with academic-related outcomes and general wellbeing among college students: the importance of measurement specificity

**DOI:** 10.1186/s40359-024-01859-7

**Published:** 2024-07-18

**Authors:** Wai-lap Lance Wong, Sing-hang Cheung

**Affiliations:** Department of Psychology, The University of Hong Kong, Jockey Club Tower, The University of Hong Kong, Pokfulam, Hong Kong

**Keywords:** Global hope, Academic hope, Academic performance, Wellbeing, Domain-generality and domain-specificity

## Abstract

**Background:**

Hope has been extensively studied as a predictor of college students’ academic success. Most previous studies used domain-general, global hope measures to gauge the association between hope and academic performance among college students. However, a few studies have suggested that hope is domain-specific and domain-specific academic hope measures should be included in related research to better assess the influence of hope on academic outcomes. In this study, we aimed to further examine this issue to ascertain if there is value in including academic hope measures when studying the link between hope and academic outcomes in college students.

**Methods:**

Two samples of Hong Kong college students (total *N* = 1321) were recruited. Each participant completed a set of self-reported online questionnaires.

**Results:**

In both samples, global hope and academic hope emerged as related but separate factors in confirmatory factor analyses. Academic hope had consistently stronger unique explanatory power on academic performance and goal setting than global hope did. On the other hand, global hope explained more variance in general wellbeing than academic hope did, but its explanatory role in academic performance was not significant.

**Conclusions:**

The findings support domain-specificity and show that hope measures explain more variance in outcomes in the matched domains. Therefore, academic hope measures should more routinely be included in related research to better evaluate the role of hope in academic pursuit among college students. Possible implications for hope interventions are also discussed.

**Supplementary Information:**

The online version contains supplementary material available at 10.1186/s40359-024-01859-7.

## Background

Academic performance is an important predictor of college students’ future success [1–4]. Plenty of studies have been conducted to examine factors that can influence college students’ academic functioning in order to find ways to enhance their academic performance. Although intellectual capability [5] and previous academic attainment [6, 7] are crucial factors in determining how much college students can achieve, there has been a growing recognition that non-intellectual dispositional factors are important in explaining academic success and failure. Among these factors, hope is an extensively studied positive psychological construct that is closely implicated in college students’ academic functioning. Snyder and his colleagues developed the widely adopted hope theory which defines hope based on goal-directed thinking [8, 9]. According to this theory, hope encompasses (1) pathway thinking in which people generate multiple strategies to achieve their goals and (2) agency thinking in which people initiate and sustain their motivations in using those strategies. People high in hope set clearer goals and are more able to find and use multiple ways to achieve their goals. When one way is impeded, they can flexibly shift to another way to continue goal pursuit [10]. Therefore, a high level of hope can facilitate goal pursuit and attainment.

Given that successful goal pursuit is central to people’s lives, hope has been found to positively influence a wide array of life outcomes, including physical and mental wellbeing [11–14]. Similar to other activities in life, academic pursuit in college is a goal-directed activity involving goal setting and finding ways to achieve goals. Hope therefore can enhance college students’ academic achievement by strengthening their ability to pursue academic-related goals. A meta-analysis by Marques et al. [15] found that hope (93% of the studies used hope measures based on Snyder’s hope theory) had an average correlation of 0.19 with academic achievement among college students. More recent studies have found the same positive linkage between hope and academic performance [16–18]. Furthermore, a handful of longitudinal studies show that a higher level of hope can prospectively predict better academic performance and outcomes in college [6, 7, 10, 19–21]. A few hope intervention programs for college students have been developed and their positive effects on promoting academic performance have been experimentally tested and demonstrated [22]. Hope also plays an important role in adaptive academic coping during emergency online learning in COVID-19 [23]. Findings from these correlational, longitudinal, experimental, and meta-analytic studies converge to support the benefit of hopeful thinking on college students’ academic pursuit.

When hope was first proposed by Snyder and his colleagues, it was conceptualized as individuals’ general sense of hope across all life domains [24]. The domain-general Dispositional Hope Scale (DHS) was developed to measure this global, generalized perception of hopeful thinking [9]. Later, the Domain-Specific Hope Scale (DSHS) was developed to tap into hope in each of the major arenas of life to better reflect hopeful thinking in specific life domains [25]. The Academic Hope Scale (AHS) is included in DSHS to measure hope specifically about academic work. Despite the development of an academic-specific hope scale, the domain-general DHS has been predominantly used to measure hopeful thinking for studying the association between hope and academic performance. The aforementioned meta-analysis by Marques et al. [15] revealed that over 84.5% of the relevant studies used domain-general instruments to measure hope. Likewise, most recent studies have adopted DHS to examine how hope is related to academic performance at different school levels [21, 26, 27]. Substantially fewer studies have used or included AHS in examining the relationship between hope and academic performance (Gallagher et al. [20] and Feldman & Kubota [28] are among a few exceptions). In fact, the choice of DHS over AHS in previous studies appears arbitrary despite the recommendations by Feldman and Kubota [28] and Robinson and Rose [29] that hope should be measured at an appropriate level of specificity. There have been few explanations regarding when and why the domain-general DHS is preferred over the domain-specific AHS when examining the association between hope and academic performance. To draw a parallel analogy, this issue is also observed when hope is studied as one of the components of psychological capital (PsyCap). In examining how PsyCap is linked to academic-related outcomes, some studies adopted domain-general PsyCap measures [30–34] while others used academic-specific PsyCap measures [35–38]. However, the reason why a particular PsyCap measure was picked or preferred over the others is usually not explained in detail.

The choice between DHS and AHS is an important issue because the two scales are likely to represent hopeful thinking in different contexts and the association between hope and academic performance of college students may differ substantially depending on which scale is used to operationalize hope. Although DHS and AHS are positively correlated, an individual’s domain-general or global hope and academic-specific hope may not always and perfectly correspond. Hope can be context-dependent and domain-specific. A high level of hope in a general context may not correspond to a high level of hope in the context of a particular life domain, and being hopeful in one life domain may not mean being hopeful about life overall. For example, Snyder et al. [10] observed that some college students who were in general hopeful scored quite low in hope in academic work. This implies a student who is able to generate and use multiple strategies to achieve life goals in general may not be equally able to do the same when it comes to academic work. This makes intuitive sense because the ability to pursue a goal probably depends on what the goal is about. For example, some students may believe they are able to pursue goals in a non-academic domain that they are interested in, but they may not think the same regarding their academic work for reasons such as a lack of passion or interest. It is possible that a student who scores high on DHS is hopeful in domains other than academic work, and this high level of global hope may therefore not necessarily make this student more capable of pursuing academic goals [39]. On the other hand, a student who scores high on AHS is clearly hopeful in academic work, which should enable him or her to perform better academically.

The distinction between DHS and AHS discussed above implies academic hope measured by AHS may have a stronger relationship with or effect on academic-related outcomes than global hope measured by DHS. The results of the meta-analysis by Marques et al. [15] provide some indirect evidence for this speculation. The average correlations of hope (mostly domain-general) with academic outcomes (ranging from 0.13 to 0.27 in absolute value) were much weaker than those with assets and liabilities which include variables pertaining to general wellbeing (ranging from 0.32 to 0.64 in absolute value). This implies the correlation is stronger when hope and outcomes are measured in a matched context (a domain-general context here). Furthermore, findings from other studies show that domain-specific hopes were more strongly related to outcomes in their respective domains [40]. The most direct evidence is provided by a handful of studies that examined DHS and AHS together in college students [28, 29]. They showed that AHS was more strongly associated with academic performance than DHS, and only AHS significantly and positively predicted academic performance while DHS was not a significant predictor when both were considered together. These support domain-specificity and suggest AHS is a more relevant hope measure and predictor for college students’ academic performance. The predictive power of DHS is likely to stem from the academic-hope component embedded in it, which, when statistically controlled, rendered DHS a non-significant predictor. Given the generally weaker correlation between DHS and academic performance, the predominant use of DHS as the hope measure in previous studies might not be able to accurately reflect the relationship between hope and academic performance of college students. There may be a need to incorporate AHS in related research to provide a better evaluation and understanding of how academic pursuit and outcomes in college are influenced by hopeful thinking.

The aforementioned matters concerning DHS and AHS can more generally be understood as an issue of domain-generality and -specificity. Some scholars have called for research efforts to address this issue because it has implications regarding how hope functions differently in different circumstances and contexts [41, 42]. In the case of DHS and AHS, if hope is domain-general, there should be a strong hope “factor” underlying both DHS and AHS (the “general-factor” model), implying that a general “hopeful outlook” is driving hopeful thinking across different contexts. In this case, the domain-general DHS may be sufficient in tapping into this generalized perception of hope and in studying how hope is related to academic pursuit. However, if hope is to a certain extent domain-specific, DHS and AHS should emerge as distinct factors (the “distinct-factor” model) in factor analyses. This would mean academic hope may not simply be a reflection of global hope and DHS may therefore not fully capture the kind of hopeful thinking specifically related to academic pursuit. In this situation, it would be necessary to include AHS when examining how hope is related to academic pursuit. The issue of domain-generality and -specificity also pertains to whether DHS and AHS would be differentially related to outcomes in different contexts. If hope is best conceptualized as being domain-general, DHS should explain a significant portion of variance in outcomes in all contexts and AHS should make little additional contributions even for academic-related outcomes. If domain-specificity is present, there should be “context-matching” explanatory power of DHS and AHS, which means they should explain outcomes in the matched context better. DHS should explain outcomes in the general context (such as general wellbeing or pursuit of life goals in general) better whereas AHS should explain more variance for outcomes specifically in the academic context. In that case, AHS would be a more relevant measure for studying how hopeful goal-directed thinking matters to academic goal pursuit of college students. Therefore, the issue of domain-generality and -specificity speaks to whether AHS should be more regularly used and incorporated in research related to hope and academics in college.

Domain-generality and -specificity have been an important topic for many psychological constructs because they have implications regarding how constructs should be conceptualized and measured. For example, this topic has been widely explored for self-efficacy [43–45], perfectionism [46–48], creativity [49–51], and grit [52, 53]. However, it has not been adequately examined for global hope and academic hope. Feldman and Kubota [28] and Robinson and Rose [29], as reviewed above, are the few studies that attempted to address this issue by studying DHS and AHS together. Their findings, as described above, support domain-specificity. However, they have caveats that make a more extensive study warranted. For Robinson and Rose [29], academic self-efficacy was not controlled when examining the predictive power of DHS and AHS on academic performance. Academic self-efficacy has been shown to be closely related to academic hope and academic performance [20]. Therefore, it is not clear whether the significant “context-matching” effect of AHS above and beyond DHS found in this study reflected the effect of academic-specific hope or academic self-efficacy. For Feldman and Kubota [28], the sample size (*N* = 89) was small which might have contributed to unclear conclusions and implications of some of the results. This study found a statistically non-significant unique direct effect of DHS on academic performance with a standardized coefficient of − 0.18. The size of this effect is quite substantial considering that it was a unique effect with many highly relevant predictors including AHS and academic self-efficacy statistically controlled. With a larger sample size, this effect of DHS might have become significant, and an unexpected negative effect of DHS might have been obtained. This would have led to the conclusion that global hope is negatively associated with academic performance when the academic-hope component has been considered. This would imply DHS and AHS could have opposite unique effects on college students’ academic performance and a more thorough consideration of how domain-general and academic-specific hopes are related to academic outcomes would be necessitated. This in turn would have implications regarding which measures should be used to operationalize hope in related research.

In addition, neither of the two studies addressed the issue of domain-generality and -specificity by directly testing whether DHS and AHS are better represented by a common hope factor or two distinct factors. Robinson and Rose [29] attempted to address this issue through exploratory factor analyses (EFA). However, the conclusion is still unclear because EFA does not provide enough information as to whether the distinct-factor model had a significantly better fit than the general-factor model. Another limitation is that both studies only examined academic performance as the outcome. While this provides information about how DHS and AHS are differentially related to academic-related outcomes, including domain-general outcomes such as general wellbeing and adjustment would give a clearer picture of the “context-matching” explanatory power of DHS and AHS through testing how the two scales are differentially related to outcomes in different situations and contexts. This would provide greater insight into the relevance of different hope measures for outcomes in different domains, and inform researchers about the choice of hope measures for different research contexts. Last but not the least, the samples of these two studies were very limited in terms of diversity since each of them only consisted of students from a psychology course in a particular college or university. A study with a more diverse sample of students from different faculties/departments and colleges is needed to better test the research questions. All in all, research into the distinction between global hope and academic hope and their domain-generality and -specificity is very scarce. Despite the recommendations by Feldman and Kubota [28] and Robinson and Rose [29], AHS has not yet been routinely incorporated in hope research related to academics. Given the above-mentioned unaddressed issues and the widespread problem of the “replication crisis” in psychological research [54], more replications and extensions in this area are needed to elucidate how hope should be conceptualized (i.e., domain-general or domain-specific) and which hope measure(s) should be included when examining the link of hope with academic performance as well as outcomes in other life domains among college students.

The main objective of the present study was to conduct a systematic examination into the issue of domain-generality and -specificity of DHS and AHS. This was aimed to further ascertain the need and value of routinely including AHS in studying the association between hope and academic-related outcomes in college students. To address the limitations of previous studies, we recruited a much larger and more diverse sample of college students than Feldman and Kubota [28] and Robinson and Rose [29], conducted confirmatory factor analyses (CFAs) to directly compare the model fit of the “general-factor” and “distinct-factor” models, and tested the “context-matching” explanatory power of DHS and AHS on academic-related outcomes as well as general wellbeing while statistically controlling for the effects of relevant covariates. Note that we recruited two independent samples of college students and conducted analyses on them separately. This serves two purposes. First, it can show the degree of replicability and consistency of results across two different samples. Second, the two samples completed different measures related to academic-related outcomes, general wellbeing, and some of the covariates. This allowed us to test the hypotheses with a wider range of variables for better generalization and replication while keeping the questionnaire short to increase the response rate.

In this study, we operationalized academic-related outcomes as (1) academic performance in terms of grade-point average (GPA) and (2) academic goal setting in terms of expected GPA [55, 56]. These two variables have been widely studied in research on hope and academic achievement [28, 57]. We chose to include academic goal setting because it has been demonstrated as an important factor in explaining the effect of hope on academic performance [21]. It also allows us to understand how global hope and academic hope may differentially influence the process whereby students formulate goals for their studies. Regarding general wellbeing, in line with Diener’s [58] definition of subjective wellbeing, we included several variables encompassing positive and negative affective/psychological states and life satisfaction. Concerning the covariates, in addition to academic self-efficacy as explained above, we also included general self-efficacy and optimism. These two variables were typically controlled in previous studies that examined the unique role of hope on academic achievement [7, 21, 29].

Based on previous findings that support domain-specificity of hope [28, 29], we hypothesized that (1) DHS and AHS would represent factorially distinct factors and the “distinct-factor” model would have a significantly better fit than the “general-factor” model, and (2) when covariates were statistically controlled, AHS would be more strongly related to academic-related outcomes than DHS while DHS would be more strongly related to general wellbeing than AHS, supporting domain-specificity and the “context-matching explanatory power” hypothesis.

## Methods

### Participants

We recruited two samples (N_sample 1_: 947, N_sample 2_: 374) of college students in Hong Kong as participants for this study. The main analyses of this study involved factor analyses, and the sample size of each of the two samples (> 300) was considered adequate for “good” factor analyses [59]. Participants of sample 1 were recruited from three local tertiary institutes. Those of sample 2 were recruited from four other institutes. Each sample consisted of students from high-tier and low-tier institutes so as to increase diversity. All participants were local college students, 18 years old or above, and competent in Chinese. The characteristics of the sample are summarized in Table 1. As an incentive for participation, one in every 20 participants who completed the questionnaire was randomly selected and awarded HKD400.

**Table 1:** Demographic characteristics of the sample

Characteristics	Sample 1 (*N* = 947)	Sample 2 (*N* = 374)
Gender	*N* (*%*)	*N* (*%*)
Male	240 (25.3)	123 (32.9)
Female	689 (72.8)	246 (65.8)
Not disclosed	18 (1.9)	5 (1.3)
Age (Years)		
18	159 (16.8)	65 (17.4)
19	185 (19.5)	71 (19.0)
20	182 (19.2)	85 (22.7)
21	195 (20.6)	71 (19.0)
22	145 (15.3)	47 (12.6)
23 or above	81 (8.6)	35 (9.4)
Family Monthly Income (HKD)		
9,999 or below	44 (4.6)	22 (5.9)
10,000 to 19,999	153 (16.2)	68 (18.2)
20,000 to 29,999	194 (20.5)	69 (18.4)
30,000 to 39,999	128 (13.5)	55 (14.7)
40,000 to 49,999	71 (7.5)	26 (7.0)
50,000 to 59,999	49 (5.2)	15 (4.0)
60,000 or above	85 (9.0)	22 (5.9)
Not sure	223 (23.5)	97 (25.9)
Year of Study		
First	296 (31.3)	95 (25.4)
Second	237 (25.0)	173 (46.3)
Third	213 (22.5)	46 (12.3)
Fourth	180 (19)	51 (13.6)
Fifth or above	21 (2.2)	9 (2.4)
Work status		
Had a full-time or part-time job	625 (66.0)	221 (59.1)
Did not have a full-time or part-time job	322 (34.0)	153 (40.9)
Religion		
Had a religion	208 (22.0)	85 (22.7)
Did not have a religion	739 (78.0)	289 (77.3)

### Measures

The two samples completed different sets of measures. Measures on demographic background, global hope, academic hope, academic self-efficacy, and general self-efficacy were completed by both samples. Sample 1 completed the Depression, Anxiety and Stress Scale – 21 items (DASS 21), Subjective Happiness Scale (SHS), and the revised Life Orientation Test (LOT). They also reported their cumulative grade-point average (CGPA). Sample 2 completed the Positive and Negative Affect Schedule (PANAS), the Satisfaction with Life Scale (SWLS), and the short form of the Beck Hopelessness Scale (BHS). They were also asked to write down their expected GPA for the current semester.

#### Demographic background

Participants answered several questions about their demographic background, including their gender, age, place of birth, family income, work status, the institute and department that they were studying in, year of study, and whether they had a religion.

#### Domain-general/global hope

The Dispositional Hope Scale (DHS) [9] was used to measure participants’ domain-general/global hope. The scale consists of 12 items. Four measure pathway thinking (e.g., “I can think of many ways to get out of a jam”), another four measure agency thinking (e.g., “I energetically pursue my goals”), and the remaining four are filler items. Each item was rated on an eight-point scale from 1 to 8. A higher score indicated a higher level of global hope.

#### Academic hope

The Academic Hope Scale (AHS) from the Domain-specific Hope Scale (DSHS) [25] was used to measure participants’ academic hope. Similar to DHS, there are four items measuring pathway thinking (e.g., “I can think of lots of ways to make good grades”) and another four items measuring agency thinking (e.g., “I energetically pursue my school work”). The items were rated on an eight-point scale from 1 to 8. A higher score indicated a higher level of academic hope.

#### Academic performance and goal setting

Participants of sample 1 were asked to report their latest CGPA as a measure of their academic performance. We used self-reported GPA because it has been shown to be very strongly correlated (*r* = .90) with actual GPA [60, 61]. Since participants were recruited from more than one institute and there might be differences in the GPA system across institutes, CGPAs were standardized within the respective institutes to account for potential between-institute differences. A higher standardized CGPA indicated better academic performance relative to other participants from the same institute.

Participants of sample 2 were asked to write down the GPA that they expected to get in the current semester as a measure of their academic goal setting. Expected GPAs were also standardized within the respective institutes, and a higher standardized expected GPA indicated that the participant expected better performance than other participants from the same institute.

#### General wellbeing

##### Affective/psychological states

Participants’ affective and psychological states were assessed by several scales. The first one is DASS 21 [62], which taps into participants’ depressive, anxiety, and stress symptoms (e.g., “I was unable to become enthusiastic about anything”). This was completed by participants of sample 1. They responded to each item by recalling their experience in the past week. The items were rated on a four-point scale from 0 to 3. Averages were calculated for the three subscales separately, and a higher average indicated more frequent experiences of the corresponding psychological state.

The second scale is SHS [63], which measures participants’ experience of happiness. This was completed by participants of sample 1. They responded to each of the four items (e.g., “In general, I consider myself…) using a seven-point scale (e.g., from 1 “not a very happy person” to 7 “a very happy person”). A higher average indicated a higher level of happiness.

The third scale is PANAS [64], which assesses participants’ positive and negative affective states. This was completed by participants of sample 2. They were presented with 20 affective states (e.g., nervous) and asked to rate how often they experienced those states on a five-point scale from 1 to 5. Averages were calculated for positive and negative affective states separately, and a higher average indicated more frequent experiences of the corresponding affective state.

##### Life satisfaction

Participants’ levels of life satisfaction were measured by SWLS [65], a five-item inventory (e.g., I am satisfied with my life) rated on a seven-point scale from 1 to 7. This was completed by participants of sample 2. A higher average indicated a greater sense of satisfaction with life.

#### Other variables

To statistically control for the effects of relevant covariates when assessing the explanatory power of DHS and AHS, the following variables were measured.

##### Academic self-efficacy

Academic self-efficacy was measured by the Academic Self-efficacy Scale developed by Chemers et al. [66]. Participants responded to eight items (e.g., “I usually do very well in school and at academic tasks”) using a seven-point scale from 1 to 7. A higher average indicated a higher level of self-efficacy in academic work.

##### General self-efficacy

General self-efficacy was measured by the General Self-efficacy Scale [67], which consists of ten items (e.g., “I can always manage to solve difficult problems if I try hard enough) rated on a four-point scale from 1 to 4. A higher average indicated a higher level of self-efficacy in general.

##### Optimism

Optimism was measured by the Chinese version of the revised LOT [68]. This was completed by participants of sample 1. Each of the six items (e.g., “In uncertain times, I always expect the best”) was rated on a five-point scale from 1 to 5. A higher average indicated a higher level of optimism.

##### Pessimism and hopelessness

Hopelessness was measured by the short form of BHS [69], which consists of four items (e.g., My future seems dark to me) rated on a six-point scale from 1 to 6. This was completed by participants of sample 2 as an alternative to optimism, which served to replicate the results using a distinct but conceptually similar covariate. A higher average indicated a greater sense of pessimism and hopelessness.

### Procedure

We obtained ethics approval for this study from the first author’s institute. Scales and inventories that are in English and do not have a Chinese version were translated into Chinese (Cantonese in traditional Chinese) using the back-translation procedure. Only the Chinese version was used in this study. Data were collected during the spring semester in 2019. Recruitment of participants was done through campus-wide emails in different local tertiary institutes. A link to the online survey of this study (administered on Qualtrics) was included in the emails. In the survey, participants first completed an informed consent form and some questions to confirm their eligibility. Those who met the inclusion criteria and consented to participate then filled out a set of questionnaires comprising the measures described above. The two samples underwent the same data collection procedure.

### Data analyses

Firstly, we conducted CFAs on the two core measures, DHS and AHS, to assess their factorial validity. Their respective one-factor structure was tested. Coefficient omega was calculated to check the internal reliability of the two scales. We also computed coefficient omega for all other scales to assess their internal reliability. Secondly, another set of CFAs were conducted to test the “general-factor model” and the “distinct-factor model” of DHS and AHS. In the first CFA, the “general-factor model” was tested by specifying the items of both scales as indicators of the same latent factor. In the second CFA, the “distinct-factor model” was tested by specifying the items of DHS as indicators of global hope and those of AHS as indicators of academic hope. Chi-squared difference tests were conducted between the two models to assess if the “distinct-factor model” had a significantly better fit than the “general-factor model”. The analyses above were conducted separately on the two samples to assess the degree of replicability of the results. In all CFAs, the diagonally weighted least square (DWLS) estimator was used because it has been suggested as a better estimator for ordinal variables (e.g., Likert-scale items) with less than nine ordered categories [70, 71]. The following was adopted as criteria for assessing model fit: Comparative Fit Indexes (CFI) > 0.95, Root Mean Square Errors of Approximation (RMSEA) < 0.05, and Standardized Root Mean Square Residual (SRMR) < 0.06 [72] indicate an acceptable model fit.

The context-matching explanatory power of DHS and AHS was then tested by path analyses in the third step. For sample 1, DHS and AHS were specified as predictors, CGPA, DASS (anxiety, depressed mood, and stress), and SHS as outcomes, and academic self-efficacy, general self-efficacy, and optimism as covariates. For sample 2, DHS and AHS were specified as predictors, expected GPA, PANAS, and SWLS as outcomes, and academic self-efficacy, general self-efficacy, and hopelessness as covariates. For both samples, institutional affiliations were also included as a covariate to statistically control for possible between-institute differences. Averages of the corresponding scales or subscales were used in the analyses and so all variables were observed variables (and the path models were therefore just identified). Since averages of multiple Likert-scale items are routinely taken as interval and continuous [73], the maximum likelihood (ML) estimator was used in the path analyses. Bootstrapping (with 1000 bootstrapped samples, using the bias-corrected and accelerated (BCa) 95% confidence intervals (CI)) was used to assess statistical significance.

Descriptive statistics of the study variables (means, SDs, and bivariate correlations) were calculated by the Statistical Package for the Social Sciences Version 26. All other analyses were conducted using the “lavaan” [74] and “MBESS” [75] packages on R.

## Results

### Factorial validity of DHS and AHS

Results from CFAs (see Table 2) showed that the one-factor structure of DHS had an acceptable model fit in both samples. The standardized factor loadings ranged from 0.49 to 0.77 and they were all statistically significant at *p* < .001. Similarly, the one-factor structure of AHS had an acceptable model fit in both samples. The standardized factor loadings were all significant at *p* < .001 and ranged from 0.68 to 0.89. The coefficient omega were 0.88 (sample 1) and 0.89 (sample 2) for DHS, and 0.93 (sample 1) and 0.94 (sample 2) for AHS. Given these, the one-factor structure of each of the two scales was supported in both samples, and DHS and AHS were hence respectively treated as unidimensional^1^ in subsequent analyses.

**Table 2:** Results of CFAs on the one-factor structure of DHS and AHS

	*X* ^*2*^	*df* (*p*)	CFI	SRMR	RMSEA	90% CI for RMSEA	
						LL	UL
DHS							
Sample 1	47.77	20 (< .001)	.993	.048	.038	.024	.052
Sample 2	11.20	20 (.941)	1.000	.036	.000	.000	.000
AHS							
Sample 1	33.72	20 (.028)	.998	.041	.027	.009	.042
Sample 2	11.95	20 (.918)	1.000	.042	.000	.000	.016

### “General-factor” vs. “distinct-factor” models

CFAs (see Table 3) revealed that the model fit of the general-factor model did not reach an acceptable level in both samples. On the other hand, the distinct-factor model had an acceptable model fit in both samples and the model fit indices were consistently better than those of the general-factor model. The chi-squared difference tests also revealed that the distinct-factor model had a significantly better model fit than the general-factor model in both samples (sample 1: Δ*X*^*2*^(1) = 456.28, *p* < .001; sample 2: Δ*X*^*2*^(1) = 96.99, *p* < .001). The inter-factor correlation between DHS and AHS was 0.71 in sample 1 and 0.79 in sample 2 (*ps* < 0.001). Consistent with our hypotheses, DHS and AHS were better conceptualized as two related but distinct factors of hope than a common, general factor. In both general-factor and distinct-factor models, all standardized factor loadings had substantial magnitudes (ranged from 0.39 to 0.86) and were statistically significant at *p* < .001 level.

**Table 3:** Results of CFAs on the “general-factor” and “distinct-factor” models

	*X* ^*2*^	*df* (*p*)	CFI	SRMR	RMSEA	90% CI for RMSEA	
						LL	UL
Sample 1							
General	678.04	104 (< .001)	.965	.091	.076	.071	.082
Distinct	221.75	103 (< .001)	.993	.052	.035	.029	.041
Sample 2							
General	171.20	104 (< .001)	.990	.074	.042	.030	.053
Distinct	74.21	103 (.985)	1.000	.048	.000	.000	.000

### Context-matching explanatory power of DHS and AHS

Descriptive statistics of the study variables, including their internal reliabilities (coefficient omega), are provided in Table 4. Their bivariate correlations are provided in Table 5. The context-matching explanatory power of DHS and AHS was tested by a series of path analyses (with covariates as mentioned above) in each sample. In the first path analysis, DHS was entered as the predictor. In the second path analysis, AHS was entered as the predictor. These were aimed at assessing the explanatory power of DHS and AHS separately. In the third path analysis, DHS and AHS were simultaneously entered as predictors. When comparing the results of the third analysis with those of the first and the second analyses, we could (1) assess the percentage of variance uniquely explained by DHS and AHS, and (2) gauge how the explanatory power of DHS or AHS changed when the other scale was added into the model. The results can be found in Table 6. For supplemental information, the results of the covariates (academic self-efficacy, general self-efficacy, and optimism/hopelessness) are provided in Appendix A.

**Table 4:** Descriptive statistics of the study variables

Variables	*M*	*SD*	ω
Sample 1			
DHS	5.61	0.98	.88
AHS	5.39	1.16	.93
Anxiety (DASS)	1.94	0.67	.87
Depressed Mood (DASS)	1.98	0.72	.90
Stress (DASS)	2.27	0.66	.84
SHS	4.18	1.17	.84
CGPA	0	1.00	/
Optimism	3.07	0.63	.71
GSE	2.64	0.53	.91
ASE	4.42	1.01	.90
Sample 2			
DHS	5.70	1.01	.89
AHS	5.40	1.21	.94
PA	3.27	0.59	.88
NA	2.97	0.74	.89
LS	4.24	1.33	.93
Expected GPA	0	1.00	/
Hopelessness	3.19	0.92	.77
GSE	2.67	0.55	.92
ASE	4.42	0.99	.90

Note. ω = Coefficient omega (internal reliability). DHS = Dispositional Hope Scale. AHS = Academic Hope Scale. SHS = Subjective happiness. GSE = General self-efficacy. ASE = Academic self-efficacy. PA = Positive Affect. NA = Negative Affect. LS = Life Satisfaction

**Table 5:** Correlations of the study variables

	1	2	3	4	5	6	7	8	9	10	11	12	13	14	15
1. DHS	**--**	.72***	-	-	-	-	-	-	.62***	− .25***	.60***	.21***	− .58***	.72***	.66***
2. AHS	.64***	**--**	-	-	-	-	-	-	.63***	− .23***	.58***	.44***	− .54***	.62***	.79***
3. Anxiety (DASS)	− .28***	− .15***	**--**	-	-	-	-	-	-	-	-	-	-	-	-
4. Depressed mood (DASS)	− .46***	− .31***	.77***	**--**	-	-	-	-	-	-	-	-	-	-	-
5. Stress (DASS)	− .25***	− .13***	.80***	.77***	**--**	-	-	-	-	-	-	-	-	-	-
6. SHS	.50***	.37***	− .42***	− .60***	− .49***	**--**	-	-	-	-	-	-	-	-	-
7. CGPA	.15***	.39***	− .06	− .08*	.03	.002	**--**	-	-	-	-	-	-	-	-
8. Optimism	.52***	.35***	− .44***	− .40***	− .59***	.67***	− .01	**--**	-	-	-	-	-	-	-
9. PA	-	-	-	-	-	-	-	**-**	--	− .10	.56***	.31***	− .57***	.58***	.70***
10. NA	-	-	-	-	-	-	-	**-**	-	--	− .33***	.10*	.50***	− .26***	− .16**
11. LS	-	-	-	-	-	-	-	**-**	-	-	--	.15**	− .56***	.49***	.60***
12. Expected GPA	-	-	-	-	-	-	-	**-**	-	-	-	--	− .16**	.16**	.43***
13. Hopelessness	-	-	-	-	-	-	-	**-**	-	-	-	-	--	− .50***	− .50***
14. GSE	.67***	.53***	− .20***	− .23***	− .34***	.45***	.04	.47***	-	-	-	-	-	**--**	.61***
15. ASE	.55***	.80***	− .12***	− .12***	− .27***	.36***	.38***	.33***	-	-	-	-	-	.49***	**--**

*p < .05. **p < .01. ***p < .001

*Note*. DHS = Dispositional Hope Scale. AHS = Academic Hope Scale. SHS = Subjective happiness. PA = Positive Affect. NA = Negative Affect. LS = Life Satisfaction. GSE = General self-efficacy. ASE = Academic self-efficacy. Correlations below the diagonal are for sample 1, and those above the diagonal are for sample 2

**Table 6:** Explanatory power of DHS and AHS

	Without the other scale		With the other scale	
	*B* (*β*)	BCa 95% CI	*B*(*β*)	BCa 95% CI
CGPA^1^				
- DHS	.08 (.08)	[-.01, .18]	− .02 (-.02)	[-.11, .06]
- AHS	.32 (.38)*	[.24, .42]	.33 (.38)*	[.24, .43]
Anxiety^1^				
- DHS	− .09 (-.13)*	[-.16, − .02]	− .09 (-.13)*	[-.16, − .01]
- AHS	− .02 (-.04)	[-.08, .04]	.01 (.01)	[-.06, .07]
Depressed Mood^1^				
- DHS	− .18 (-.25)*	[-.24, − .13]	− .17 (-.23)*	[-.23, − .11]
- AHS	− .07 (-.12)*	[-.13, − .02]	− .02 (-.03)	[-.08, .04]
Stress^1^				
- DHS	− .05 (-.08)	[-.12, .01]	− .05 (-.08)	[-.12, .01]
- AHS	.01 (.03)	[-.04, .08]	.03 (.06)	[-.03, .10]
SHS^1^				
- DHS	.17 (.14)*	[.07, .26]	.17 (.14)*	[.07, .26]
- AHS	.03 (.03)	[-.06, .12]	− .03 (-.03)	[-.13, .07]
Expected GPA^2^				
- DHS	− .04 (-.04)	[-.21, .11]	− .17 (-.18)*	[-.32, − .02]
- AHS	.34 (.42)*	[.20, .48]	.39 (.47)*	[.25, .54]
Positive Affect^2^				
- DHS	.07 (.13)*	[.01, .14]	.07 (.11)*	[.003, .13]
- AHS	.04 (.08)	[-.02, .10]	.02 (.05)	[-.05, .09]
Negative Affect^2^				
- DHS	.06 (.09)	[-.04, .17]	.06 (.08)	[-.05, .15]
- AHS	.04 (.06)	[-.05, .13]	.02 (.04)	[-.07, .13]
Life Satisfaction^2^				
- DHS	.33 (.25)*	[.16, .52]	.31 (.24)*	[.13, .50]
- AHS	.14 (.13)	[-.01, .30]	.06 (.05)	[-.08, .22]

*Note*. DHS = Dispositional Hope Scale. AHS = Academic Hope Scale. SHS = Subjective happiness.

* indicates statistically significant results based on bootstrapping (*α* = 0.05).

^1^ and ^2^ denote results based on sample 1 and sample 2 respectively.

Statistical results of “Without the other scale” are from the first (DHS only) and second (AHS only) path analyses, and those of “With the other scale” are from the third path analysis (DHS and AHS together)

In sample 1, the first path analysis revealed that DHS significantly and negatively predicted anxiety and depressed mood and positively predicted SHS. However, it did not significantly predict CGPA and stress. In the second path analysis, AHS significantly and positively predicted CGPA and negatively predicted depressed mood, but its explanatory role on SHS, anxiety, and stress was not significant. In the third path analysis, DHS still significantly predicted anxiety, depressed mood, and SHS in the presence of AHS. On the other hand, AHS still significantly predicted CGPA in the presence of DHS, but its explanatory role on depressed mood became non-significant. The percentage of variance uniquely explained by DHS was < 0.1% for CGPA, 0.9% for SHS, 0.3% for stress, 0.7% for anxiety, and 2.3% for depressed mood, and that by AHS was 4.3% for CGPA, 0.1% for SHS and stress respectively, and < 0.1% for anxiety and depressed mood respectively.

In sample 2, the first path analysis indicated that DHS significantly and positively predicted positive affect and life satisfaction but not expected GPA and negative affect. The second path analysis showed that AHS significantly and positively predicted expected GPA but not positive affect, negative affect, and life satisfaction. In the third path analysis, DHS still significantly predicted positive affect and life satisfaction in the presence of AHS. Surprisingly, when AHS had been considered, DHS negatively predicted expected GPA. On the other hand, AHS still significantly predicted expected GPA in the presence of DHS. The percentage of variance uniquely explained by DHS was 1% for expected GPA, 0.4% for positive affect, 0.2% for negative affect, and 1.9% for life satisfaction, and that by AHS was 6.3% for expected GPA, 0.1% for positive affect and life satisfaction respectively, and < 0.1% for negative affect^2^.

We also computed the variance inflation factors (VIFs) to assess if there was excessive multicollinearity among global hope, academic hope, and the covariates. The VIFs ranged from 1.43 to 3.34 in sample 1 and from 1.60 to 3.33 in sample 2. Since all VIFs were below the conventional threshold of 5, there was no concern for excessive multicollinearity [76].

In sum, when optimism (or hopelessness), general self-efficacy, academic self-efficacy, and institutional affiliations had been statistically controlled, AHS predicted academic-related variables more strongly than DHS, while its explanatory power on general wellbeing was much weaker and became non-significant when DHS had been considered. On the other hand, when the same covariates were statistically controlled, DHS predicted a range of general wellbeing indicators more strongly than AHS. Its explanatory power on CGPA was not significant, and, surprisingly, it became a negative predictor of expected GPA when AHS had been considered. Overall, the results supported our hypotheses regarding the context-matching explanatory power of DHS and AHS.

## Discussion

Across two college student samples, our results showed that DHS and AHS were better conceptualized as two related but distinct factors than a global, general factor. This is consistent with our first hypothesis and supports the notion that hope is to a certain extent domain-specific. Global hope and academic hope can be differentiated, and they represent hopeful thinking in different contexts (a general vs. an academic context here) [29, 40]. Path analyses provided support to our second hypothesis regarding the “context-matching” explanatory power of DHS and AHS. When relevant covariates were statistically controlled, DHS predicted general wellbeing better than AHS, while AHS was a more relevant predictor of academic-related variables than DHS. DHS significantly predicted various indicators of general wellbeing in the expected direction when examined alone and with AHS, while AHS significantly and positively predicted academic performance and goal setting when examined alone and with DHS. On the other hand, AHS significantly predicted depressed mood when examined alone, but this explanatory power became non-significant when DHS had been considered. DHS was not significantly related to academic performance and goal setting when examined alone, and it became a significant negative predictor of academic goal setting when AHS had been considered. In the following sections, we will explain the reasons and implications of these patterns of results.

### DHS and AHS as distinct hope factors

When comparing the “general-factor” and “distinct-factor” models, the latter had a significantly better model fit in both samples. This suggests DHS and AHS should be regarded as separate factors in college students. The former captures one’s global and generalized perceptions of pathway and agency across all life domains [9], while the latter denotes one’s pathway and agency thinking specifically about academic work and studies [25]. This is in line with the idea that hope is to some extent domain-specific and hopeful thinking pertaining to academics is related to but not simply a reflection of global, domain-general hope. These results are consistent with Robinson and Rose [29]. Our study extends their findings by statistically showing the “distinct-factor” model as significantly better than the “general-factor” model. It should be noted that DHS and AHS were strongly correlated (*r* ranged from 0.71 to 0.79). This implies the correspondence between global hope and academic hope was quite high, so college students’ levels of global hope and academic hope are fairly consistent. Despite this, there is still substantial non-shared variance (∼ 36–50%) between DHS and AHS and so the consistency is not absolute. This matches the observation of Snyder et al. [10] that students scoring high in hope in general could be quite low in hope about their academic work. Together with the findings that DHS and AHS represented separate latent factors, these imply DHS may not fully capture the kind of hope that is specific to academic studies. AHS, given its focus on academic goal-directed thinking, may be a more appropriate measure in research related to how hope matters to academic pursuit and performance in college.

### Explanatory power of DHS and AHS on academic-related variables and general wellbeing

Our results regarding the “context-matching” explanatory power of the two scales provide additional support for incorporating AHS in studying the relationship between hope and academic-related variables among college students. With relevant covariates statistically controlled, AHS had significant unique contributions in explaining the variance of academic performance and goal setting when it was examined alone and with DHS. DHS, however, had no significant unique explanatory power on academic performance no matter whether it was examined alone or with AHS. This suggests AHS is a more relevant and stronger predictor of academic performance than DHS. This is consistent with our hypothesis and findings in previous studies that AHS is more strongly associated with academic performance than DHS [28, 29]. On the other hand, DHS had significant and stronger explanatory power on general wellbeing than AHS when it was examined alone and with AHS. These patterns of results support our hypotheses and the idea that hope measures predict outcomes in the matched contexts more strongly. Given these, the predominant use of DHS in studying the association between hope and academic-related variables among college students in previous studies could have underestimated the strength of the role of hope in academic performance and pursuit. Future research should consider more thoroughly the choice of hope measures in related research. It may be beneficial to more routinely include or incorporate AHS in studying how hope is related to or influences academic-related variables among college students given its specific focus on and stronger relationship with academics. On the other hand, for research that focuses on the role of hope in non-academic and more domain-general outcomes such as subjective wellbeing and general adjustment, DHS would be a better choice given its stronger explanatory power on these variables as demonstrated in this study.

There are two additional points that are worth mentioning. The first one is the finding that DHS was not significantly associated with academic performance when examined alone or with AHS. No matter whether the academic-hope component was partialed out or not, global hope explained very little variance in academic performance. This is contrary to previous findings that DHS was a significant predictor of college students’ academic performance [6, 7, 10, 19]. This inconsistency can possibly be explained by the inclusion of relevant covariates in our study, particularly academic self-efficacy. Previous studies that found a significant association between global hope and academic performance in college students typically did not statistically control for the effect of academic self-efficacy [6, 7, 10, 19]. Self-efficacy is closely related to hope in the way that both are positive expectancy constructs [10]. Academic self-efficacy is about positive expectations of successfully completing academic tasks [77]. Given their similarity, it is possible that the significant association between global hope and academic performance found in previous studies was due to academic self-efficacy [20]. Hence, in our study, when academic self-efficacy was statistically controlled, it rendered the explanatory role of global hope non-significant. It should also be noted that, unlike global hope, academic hope remained a significant predictor of academic performance even when academic self-efficacy was statistically controlled. These results have two important implications. First, it is necessary to include appropriate covariates so that the unique effect of hope above and beyond other similar constructs can be reasonably determined. Second, academic hope measured by AHS may have better unique utility than global hope measured by DHS in predicting academic performance beyond other similar positive expectancy constructs. This echoes our earlier conclusion that incorporating AHS in related research would be useful, especially when researchers want to gauge the unique effect of hope on academic-related variables.

The second point is that DHS was found to be a negative predictor of academic goal setting when AHS had been considered. At the zero-order level, DHS was positively associated with expected GPA (*r* = .21). However, when AHS had been statistically controlled, DHS negatively predicted students’ expected GPA. Although this contradicts most of the previous findings that global hope predicted better expected academic performance [21, 57], it is consistent with Feldman and Kubota [28] which found a non-significant but substantial negative direct effect of global hope on GPA (when academic hope had been considered). One possible explanation is that the positive explanatory power of global hope found in previous studies stems from the academic-hope component embedded in it. When this component has been considered (by including AHS as an additional predictor), the unique relationship between global hope and expected GPA probably represents the relationship between non-academic hope and academic goal setting. It is possible that college students who have higher hope in non-academic domains put more effort in these domains than in academic work, so they may not expect very good performance in their studies and therefore set a lower goal for their academic work. In short, global hope is likely to include a mix of academic and non-academic hopes [39]. The academic-hope component has a more consistent positive influence on academic pursuit, but the non-academic component may have a different and unexpected effect. These contrasting effects are probably one of the reasons why global hope overall has a substantially weaker explanatory power on academic-related variables. These again highlight the importance of including hope measures at an appropriate level of specificity for uncovering a more consistent relationship between hope and academic pursuit. Incorporating AHS would be necessary to achieve this and to differentiate between the effects of academic hope and non-academic hope on college students’ academic pursuit.

### Implications

As discussed in the introduction section, research on domain-generality and -specificity of global hope and academic hope has been extremely scarce. The predominant use of domain-general DHS in gauging the association between hope and academic performance among college students also renders it unclear as to whether there is value in including domain-specific AHS in related research. This study is one among the very few that attempt to address these issues. By addressing the limitations in previous studies, our findings provide further empirical support for domain-specificity of hope and demonstrate that there is value in regularly incorporating AHS in research on the link between hope and academic pursuit in college students. AHS, which has stronger and more consistent positive associations with college students’ academic performance and goal setting than DHS, should more routinely be included in related research to better elucidate and estimate the strength of the influence of hope on college students’ academic pursuit.

It should be noted that we are not advocating exclusively using AHS. Some studies may investigate the relationship between hope and outcomes in different domains and academic pursuit may only be one of these domains (e.g., Rand et al. [21], which investigated academic performance and subjective wellbeing as outcomes). As our study shows, hope measures have “context-matching” explanatory power and so DHS is also needed if domain-general outcomes such as general wellbeing are among the outcomes that are being studied. In addition, some studies may conceptualize global hope as a more distal predictor of academic performance whose influence is mediated by academic hope [28]. Clearly, AHS and DHS should both be used in these two cases. What we would like to advocate is to regularly include academic hope measures in addition to global hope measures in related research. This allows researchers to measure hope at a level appropriate for its respective outcomes. Our suggestion is in line with those by Feldman and Kubota [28] and Robinson and Rose [29], and we hope the findings from this study will encourage researchers to consider more thoroughly the hope measures to be used in related studies. It would also be desirable if researchers routinely justify the choice of hope measures used in their studies so that the reason why a certain hope measure is preferred over the others is made clear. All in all, measuring hope at a level appropriate for its respective outcome would offer a better understanding of how hope influences outcomes in different life domains.

Our findings also have potential implications regarding how hope interventions should be conducted. A few hope intervention programs for college students have been developed [22, 78]. They tend to target pathway and agency thinking regarding general goal pursuit and aim at enhancing global hope. These programs are expected to work well with general wellbeing. However, if the aim is to promote academic performance, it may be necessary to focus on pathway and agency thinking specifically concerning academic work because, as suggested by our findings, enhancing academic hope would work better with academic performance. Similarly, if the aim is to promote functioning in multiple domains, the programs would work better if elements to enhance global hope as well as hope in specific domains are included. In short, we anticipate that hope interventions would be more effective if they are administered at a level specific enough and appropriate for their intended outcomes. Further research should be conducted to verify this speculation.

### Limitations and directions for future research

Despite the contributions, this study has several caveats that warrant attention. First, although the results support the context-matching explanatory power of global hope and academic hope, the amount of variance explained is quite small. Future research should explore factors that may affect the strength of hope’s explanatory power. It will also be necessary to compare hope’s effect with those of other predictors to assess whether hope would play a more or less important role in explaining individual variations in wellbeing and academic pursuit. Second, we focused only on college students. Hence, it is not known whether the distinction between global hope and academic hope and their “context-matching” explanatory power would be generalizable to students at other school levels. Robinson and Rose [29] suggested that the perception of hope may be more differentiated when students enter college. This is probably because the life of college students is more diversified and their perceptions of different life domains may differ more. On the other hand, for elementary and secondary school students, academic work is likely the most important part of their life and their perceptions may center on this domain more. Therefore, the correspondence between global hope and academic hope may be stronger for these students. Considering these, it is necessary to replicate this study in elementary and secondary school students to examine the developmental trend of the differentiation of global hope and academic hope.

Third, we analyzed global hope and academic hope as unidimensional constructs. This was done considering their satisfactory one-factor structure and the high correlations between the pathway and agency components. Nonetheless, this precluded us from examining the differential explanatory power of the two components. Future research should adopt a different conceptualization of hope and explore the effects of the two components separately. Fourth, this study was conducted in a Chinese context, so the findings may not be generalizable to college students in other cultures. Subsequent research should replicate this study in students of other ethnicities and cultural backgrounds. Fifth, although we statistically controlled for the effects of various relevant covariates, there are other possible confounding variables, such as parental expectations which are known to influence students’ academic pursuit and wellbeing [79, 80], that need to be considered in future studies. Lastly, this study is a cross-sectional study, so it remains unclear whether hope is an antecedent or outcome of academic performance and wellbeing. Longitudinal research should be conducted to better understand how hope may predict college students’ academic pursuit and adjustment and how the level of hope may fluctuate over time.

### Electronic supplementary material

Below is the link to the electronic supplementary material.

Supplementary Material 1

## Data Availability

Data files associated with this study are available upon reasonable request.

## Footnotes

1 We also examined the two-factor structure of DHS and AHS. The results indicated very high correlations (ranging from 0.86 to 0.97) between the pathway and agency components. This further justified our choice of treating the two hope constructs as unidimensional.

2 We reran the analyses using structural equation modelling in which DHS, AHS, and the general wellbeing variables were modelled as latent variables with their respective items as indicators (CGPA and expected GPA, as well as the covariates, were modelled as observed variables). Although the overall model fit indices did not reach a satisfactory level (which was likely due to the less-than-optimal factor structure of some of the general wellbeing variables), the results pertaining to the path coefficients were mostly the same as those from the path analyses. When DHS and AHS were considered together, the former significantly predicted general wellbeing while the latter did not. On the other hand, AHS significantly and positively predicted CGPA and expected GPA, while DHS significantly and negatively predicted expected GPA.

## References

[1] Arbona C. The development of academic achievement in school aged children: precursors to career development. In: Brown SD, Lent RW, editors. Handbook of counselling psychology. Wiley; 2000. pp. 270–309.

[2] Kool A, Mainhard MT, Jaarsma ADC, Brekelmans M, Beukelen P (2016). Academic success and early career outcomes: can honors alumni be distinguished from non-honors alumni?. High Ability Stud.

[3] Kuncel NR, Hezlett SA, Ones DS (2004). Academic performance, career potential, creativity, and job performance: can one construct predict them all?. J Personal Soc Psychol.

[4] Kuncel NR, Ones DS, Sackett PR (2010). Individual differences as predictors of work, educational, and broad life outcomes. Pers Indiv Differ.

[5] Kaufman AS, Lichtenberger EO. Assessing adolescent and adult intelligence. Wiley; 2005.

[6] Day L, Hanson K, Maltby J, Proctor C, Wood A (2010). Hope uniquely predicts objective academic achievement above intelligence, personality, and previous academic achievement. J Res Pers.

[7] Rand KL, Martin AD, Shea AM (2011). Hope, but not optimism, predicts academic performance of law students beyond previous academic achievement. J Res Pers.

[8] Snyder CR (1989). Reality negotiation: from excuses to hope and beyond. J Soc Clin Psychol.

[9] Snyder CR, Harris C, Anderson JR, Hollersan SA, Irving LM, Sigmon ST, Yoshinobu L, Gibb J, Langelle C, Hamey P (1991). The will and the ways: development and validation of an individual-differences measure of hope. J Personal Soc Psychol.

[10] Snyder CR, Shorey HS, Cheavens J, Pulvers KM, Adams III, Wiklund C (2002). Hope and academic success in college. J Educ Psychol.

[11] Counted V, Pargament KI, Bechara AO, Joynt S, Cowden RG (2022). Hope and well-being in vulnerable contexts during the COVID-19 pandemic: does religious coping matter?. J Posit Psychol.

[12] Rasmussen HN, England E, Cole BP. Hope and physical health. Curr Opin Psychol. 2022;Advance online publication. 10.1016/j.copsyc.2022.101549. 10.1016/j.copsyc.2022.101549 36640676

[13] Werner S (2012). Subjective well-being, hope, and needs of individuals with serious mental illness. Psychiatry Res.

[14] Yildirim M, Arslan G (2022). Exploring the associations between resilience, dispositional hope, preventive behaviors, subjective well-being, and psychological health among adults during early stage of COVID-19. Curr Psychol.

[15] Marques SC, Gallagher MW, Lopez SJ (2017). Hope- and academic-related outcomes: a meta-analysis. School Mental Health.

[16] Ge JL, Feldman DB, Shu TM (2023). The relationships of hope, optimism, and academic motivation with GPA among university students in Hong Kong. Psychol Rep.

[17] Tomas JM, Gutierrez M, Georgieva S, Hernandez M (2020). The effects of self-efficacy, hope, and engagement on the academic achievement of secondary education in the Dominican Republic. Psychol Sch.

[18] Zeinalipour H (2022). School connectedness, academic self-efficacy, and academic performance: mediating role of hope. Psychol Rep.

[19] Bryce CI, Fraser AMJ, Fabes RA, Alexander BL (2021). The role of hope in college retention. Learn Individual Differences.

[20] Gallagher MW, Marques SC, Lopez SJ (2017). Hope and the academic trajectory of college students. J Happiness Stud.

[21] Rand KL, Shanahan ML, Fischer IC, Fortney SK (2020). Hope and optimism as predictors of academic performance and subjective well-being in college students. Learn Individual Differences.

[22] Davidson OB, Feldman DB, Margalit M (2012). A focused intervention for 1st-year college students: promoting hope, sense of coherence, and self-efficacy. J Psychol.

[23] Wong WL, Yuen KA (2023). Online learning stress and Chinese college students’ academic coping during COVID-19: the role of academic hope and academic self-efficacy. J Psychol.

[24] Snyder CR. The psychology of hope: you can get there from here. Free; 1994.

[25] Sympson S. (1999). Validation of the domain specific hope scale: Exploring hope in life domains. Unpublished doctoral dissertation. Lawrence: University of Kansas.

[26] Chen J, Huebner ES, Tian L (2020). Longitudinal relations between hope and academic achievement in elementary school students: behavioral engagement as a mediator. Learn Individual Differences.

[27] Fraser AM, Bryce CI, Alexander BL, Fabes RA (2021). Hope levels across adolescence and the transition to high school: associations with school stress and achievement. J Adolesc.

[28] Feldman DB, Kubota M (2015). Hope, self-efficacy, optimism, and academic achievement: distinguishing constructs and levels of specificity in predicting college grade-point average. Learn Individual Differences.

[29] Robinson C, Rose S (2010). Predictive, construct, and convergent validity of general and domain-specific measures of hope for college student academic achievement. Res Schools.

[30] Chen P, Lin C, Lin I, Lo C (2022). The mediating effects of psychological capital and academic self-efficacy on learning outcomes of college freshmen. Psychol Rep.

[31] Kornas-Biela D, Martynowska K, Zysberg L (2020). Faith conquers all? Demographic and psychological resources and their associations with academic performance among religious college students. Br J Religious Educ.

[32] Siu OL, Bakker AB, Jiang X (2014). Psychological capital among university students: relationships with study engagement and intrinsic motivation. J Happiness Stud.

[33] Wang J, Bu L, Li Y, Song J, Li N (2021). The mediating effect of academic engagement between psychological capital and academic burnout among nursing students during the COVID-19: a cross-sectional study. Nurse Educ Today.

[34] Zhang C, Li G, Fan Z, Tang X, Zhang F (2021). Psychological capital mediates the relationship between problematic smartphone use and learning burnout in Chinese medical undergraduates and postgraduates: a cross-sectional study. Front Psychol.

[35] Datu JAD, King RB, Valdez JPM (2018). Psychological capital bolsters motivation, engagement, and achievement: cross-sectional and longitudinal studies. J Posit Psychol.

[36] Liran BH, Miller P (2019). The role of psychological capital in academic adjustment among university students. J Happiness Stud.

[37] Luthans BC, Luthans KW, Jensen SM (2012). The impact of business school students’ psychological capital on academic performance. J Educ Bus.

[38] Martinez IM, Youssef-Morgan CM, Chambel MJ, Marques-Pinto A (2019). Antecedents of academic performance of university students: academic engagement and psychological capital resources. Educational Psychol.

[39] Shorey HS, Roberts CRD, Huprich SK (2012). The roles of domain specific hope and depressive personality in predicting depressive symptoms. Personality Mental Health.

[40] Kwon P (2002). Hope, defense mechanisms, and adjustment: implications for false hope and defensive hopelessness. J Pers.

[41] Campbell DG, Kwon P (2001). Domain-specific hope and personal style: toward an integrative understanding of dysphoria. J Soc Clin Psychol.

[42] Chang EC. Hope, problem-solving ability, and coping in a college student population: some implications for theory and practice. J Clin Psychol. 1998;54:953–62. 10.1002/(SICI)1097-4679(199811)54:7%3C953::AID-JCLP9%3E3.0.CO;2-F. 10.1002/(sici)1097-4679(199811)54:7<953::aid-jclp9>3.0.co;2-f 9811132

[43] Ehrenberg MF, Cox DN, Koopman RF (1991). The relationship between self-efficacy and depression in adolescents. Adolescence.

[44] Grether T, Sowislo JF, Wiese BS (2018). Top-down or bottom-up: prospective relations between general and domain-specific self-efficacy beliefs during a work-family transition. Pers Indiv Differ.

[45] Pintrich PR, Schunk DH. Motivation in education: theory, research, and applications. 2nd ed. Prentice Hall; 2002.

[46] Haase AM, Prapavessis H, Owens RG (2013). Domain-specificity in perfectionism: variations across domains of life. Pers Indiv Differ.

[47] Levine SL, Milyavskaya M (2018). Domain-specific perfectionism: an examination of perfectionism beyond the trait-level and its link to well-being. J Res Pers.

[48] Stoeber J, Stoeber FS (2009). Domains of perfectionism: prevalence and relationships with perfectionism, gender, age, and satisfaction with life. Pers Indiv Differ.

[49] Han K, Marvin C (2002). Multiple creativities? Investigating domain-specificity of creativity in young children. Gifted Child Q.

[50] Qian M, Plucker JA, Yang X (2019). Is creativity domain specific or domain general? Evidence from multilevel explanatory item response theory models. Think Skills Creativity.

[51] Sternberg RJ. Domain-generality versus domain-specificity of creativity. In: Meusburger P, Funke J, Wunder E, editors. Milieus of creativity: an interdisciplinary approach to spatiality of creativity. Volume 2. Springer; 2009. pp. 25–38.

[52] Cormier DL, Dunn JGH, Dun JC (2019). Examining the domain specificity of grit. Pers Indiv Differ.

[53] Schmidt FTC, Fleckenstein J, Retelsdorf J, Eskreis-Winkler L, Moller J (2019). Measuring grit: a German validation and a domain-specific approach to grit. Eur J Psychol Assess.

[54] Shrout PE, Rodgers JL (2018). Psychology, science, and knowledge construction: broadening perspectives from the replication crisis. Ann Rev Psychol.

[55] Richardson M, Abraham C, Bond R (2012). Psychological correlates of university students’ academic performance: a systematic review and meta-analysis. Psychol Bull.

[56] Zimmerman BJ, Bandura A, Martinez-Pons M (1992). Self-motivation for academic attainment: the role of self-efficacy beliefs and personal goal setting. Am Educ Res J.

[57] Rand KL (2009). Hope and optimism: latent structures and influences on grade expectancy and academic performance. J Pers.

[58] Diener E (1994). Assessing subjective well-being: Progress and opportunities. Soc Indic Res.

[59] Comrey AL, Lee HB. A first course in factor analysis. 2nd ed. Lawrence Erlbaum Associates, Inc; 1992.

[60] Cassady JC. Self-reported GPA and SAT scores. Eric Digest; 2001.

[61] Kuncel NR, Crede M, Thomas LL (2005). The validity of self-reported grade point averages, class ranks, and test scores: a meta-analysis and review of the literature. Rev Educ Res.

[62] Henry JD, Crawford JR (2005). The short-form version of the Depression anxiety stress scales (DASS-21): construct validity and normative data in a large non-clinical sample. Br J Clin Psychol.

[63] Lyubomirsky S, Lepper HS (1999). A measure of subjective happiness: preliminary reliability and construct validation. Soc Indic Res.

[64] Watson D, Clark LA, Tellegen A (1988). Development and validation of brief measures of positive and negative affect: the PANAS scales. J Personal Soc Psychol.

[65] Diener E, Emmons RA, Larsen RJ, Griffin S (1985). The satisfaction with Life Scale. J Pers Assess.

[66] Chemers MM, Hu L, Garcia BF (2001). Academic self-efficacy and first year college student performance and adjustment. J Educ Psychol.

[67] Schwarzer R, Babler J, Kwiatek P, Schroder K, Zhang JX (1997). The assessment of optimistic self-beliefs: comparison of the German, Spanish, and Chinese versions of the general self-efficacy scale. Appl Psychol.

[68] Lai JCL, Yue X (2000). Measuring optimism in Hong Kong and mainland Chinese with the revised life orientation test. Pers Indiv Differ.

[69] Yip PSF, Cheung YB. (2006). Quick assessment of hopelessness: a cross-sectional study. Health and Quality of Life outcomes, 4, Article number: 13. 10.1186/1477-7525-4-13. 10.1186/1477-7525-4-13 PMC1413514 16509984

[70] Li C (2016). The performance of ML, DWLS, and ULS estimation with robust corrections in structural equation models with ordinal variables. Psychol Methods.

[71] Sellbom M, Tellegen A (2019). Factor analysis in psychological assessment research: common pitfalls and recommendations. Psychol Assess.

[72] Hu L, Bentler PM (1999). Cutoff criteria for fit indexes in covariance structure analysis: conventional criteria versus new alternatives. Struct Equ Model.

[73] Hatcher L. Advanced statistics in research: Reading, understanding, and writing up data analysis results. Shadow Finch Media LLC; 2013.

[74] Rosseel Y (2012). Lavaan: an R package for structural equation modeling. J Stat Softw.

[75] Kelley K. (2017). *MBESS* (Version 4.0.0 and higher) [computer software and manual]. Accessible from http://cran.r-project.org.

[76] Hair JF, Ringle CM, Sarstedt M (2011). PLS-SEM: indeed a silver bullet. J Mark Theory Pract.

[77] Schunk DH, DiBenedetto MK. Academic self-efficacy. In: Furlong MJ, Gilman R, Huebner ES, editors. Handbook of positive psychology in schools. Routledge; 2014. pp. 115–30.

[78] Feldman DB, Dreher DE (2012). Can hope be changed in 90 minutes? Testing the efficacy of a single-session goal-pursuit intervention for college students. J Happiness Stud.

[79] Wang L, Heppner PP (2002). Assessing the impact of parental expectations and psychological distress on Taiwanese college students. Couns Psychol.

[80] Ma Y, Siu A, Tse WS (2018). The role of high parental expectations in adolescents’ academic performance and depression in Hong Kong. J Fam Issues.

