# Supplementary material for: Hope and its associations with academic-related outcomes and general wellbeing among college students: the importance of measurement specificity
Source: BMC Psychol. 2024 Jul 18;12:398. doi: 10.1186/s40359-024-01859-7 (PMC11256503; doi:10.1186/s40359-024-01859-7)
Supplement: Supplementary file 1 — Supplementary Material 1 [file 40359_2024_1859_MOESM1_ESM.docx]

**Appendix A**

|  | GSE | ASE | Optimism | Hopelessness |
| --- | --- | --- | --- | --- |
|  | *B* (*β*) | *B*(*β*) | *B*(*β*) | *B*(*β*) |
| CGPA^1^ |  |  |  |  |
| - With DHS only  - With AHS only | -.33 (-.17)*  -.37 (-.20)* | .44 (.46)*  .21 (.21)* | -.20 (-.13)*  -.20 (-.12)* | -  - |
| - With DHS and AHS | -.36 (-.19)* | .21 (.21)* | -.19 (-.12)* | - |
| Anxiety^1^ |  |  |  |  |
| - With DHS only  - With AHS only  - With DHS and AHS | -.03 (-.02)  -.09 (-.07)  -.03 (-.02) | .05 (.08)  .05 (.07)  .05 (.07) | -.36 (-.34)*  -.39 (-.37)*  -.36 (-.34)* | -  -  - |
| Depressed Mood^1^ |  |  |  |  |
| - With DHS only  - With AHS only  - With DHS and AHS | .04 (.03)  -.08 (-.06)  .04 (.03) | -.01 (-.01)  .01 (.01)  .01 (.01) | -.50 (-.44)*  -.56 (-.49)*  -.50 (-.44)* | -  -  - |
| Stress^1^ |  |  |  |  |
| - With DHS only  - With AHS only  - With DHS and AHS | .03 (.02)  -.02 (-.02)  .02 (.02) | .04 (.06)  .01 (.02)  .01 (.02) | -.44 (-.42)*  -.46 (-.44)*  -.44 (-.42)* | -  -  - |
| SHS^1^ |  |  |  |  |
| - With DHS only  - With AHS only  - With DHS and AHS | .17 (.08)*  .30 (.13)*  .17 (.08)* | .08 (.07)*  .10 (.09)*  .10 (.09)* | 1.02 (.54)*  1.08 (.57)*  1.02 (.54)* | -  -  - |
| Expected GPA^2^ |  |  |  |  |
| - With DHS only  - With AHS only  - With DHS and AHS | -.21 (-.12)  -.38 (-.21)*  -.26 (-.14)* | .55 (.54)*  .28 (.28)*  .30 (.29)* | -  -  - | .03 (.03)  .11 (.10)  .08 (.08) |
| Positive Affect^2^ |  |  |  |  |
| - With DHS only  - With AHS only  - With DHS and AHS | .12 (.11)*  .16 (.15)*  .12 (.11)* | .26 (.43)*  .25 (.41)*  .24 (.41)* | -  -  - | -.14 (-.22)*  -.15 (-.24)*  -.14 (-.22)* |
| Negative Affect^2^ |  |  |  |  |
| - With DHS only  - With AHS only  - With DHS and AHS | -.23 (-.17)*  -.20 (-.15)*  -.23 (-.17)* | .12 (.16)*  .11 (.14)  .10 (.14) | -  -  - | .45 (.56)*  .44 (.55)*  .45 (.56)* |
| Life Satisfaction^2^ |  |  |  |  |
| - With DHS only  - With AHS only  - With DHS and AHS | .07 (.03)  .28 (.12)  .06 (.03) | .39 (.29)*  .38 (.28)*  .36 (.26)* | -  -  - | -.38 (-.26)*  -.42 (-.30)*  -.37 (-.26)* |

*Effects of Covariates.*

*Note.* * indicates statistically significant results based on bootstrapping (*α* = .05).

DHS = Dispositional Hope Scale. AHS = Academic Hope Scale. GSE = General self-efficacy. ASE = Academic self-efficacy. SHS = Subjective happiness

^1^ and ^2^ denote results based on sample 1 and sample 2 respectively.

Statistical results of “With DHS only” and “With AHS only” are from the first (DHS only) and second (AHS only) path analyses, and those of “With DHS and AHS” are from the third path analysis (DHS and AHS together).
